# Physical Approaches to Prevent and Treat Bacterial Biofilm

**DOI:** 10.3390/antibiotics12010054

**Published:** 2022-12-29

**Authors:** Alexa A. Ciarolla, Norman Lapin, Dustin Williams, Rajiv Chopra, David E. Greenberg

**Affiliations:** 1 School of Medicine, University of Texas Southwestern Medical Center, Dallas, TX 75390, USA; 2 Department of Radiology, UT Southwestern Medical Center, Dallas, TX 75390, USA; 3 Department of Microbiology and Immunology, University of Utah School of Medicine, Salt Lake City, UT 84112, USA; 4 Department of Microbiology, University of Texas Southwestern Medical Center, Dallas, TX 75390, USA; 5 Department of Internal Medicine, Infectious Diseases and Geographic Medicine, University of Texas Southwestern Medical Center, Dallas, TX 75390, USA

**Keywords:** biofilm treatment, biofilm removal, prosthetic joint infection, non-pharmacologic methods, physical energy

## Abstract

Prosthetic joint infection (PJI) presents several clinical challenges. This is in large part due to the formation of biofilm which can make infection eradication exceedingly difficult. Following an extensive literature search, this review surveys a variety of non-pharmacological methods of preventing and/or treating biofilm within the body and how they could be utilized in the treatment of PJI. Special attention has been paid to physical strategies such as heat, light, sound, and electromagnetic energy, and their uses in biofilm treatment. Though these methods are still under study, they offer a potential means to reduce the morbidity and financial burden related to multiple stage revisions and prolonged systemic antibiotic courses that make up the current gold standard in PJI treatment. Given that these options are still in the early stages of development and offer their own strengths and weaknesses, this review offers an assessment of each method, the progress made on each, and allows for comparison of methods with discussion of future challenges to their implementation in a clinical setting.

## 1. Introduction

The formation of biofilm is a major pathogenic mechanism that results in antibiotic tolerant infections. This is particularly true for patients with medical implants such as catheters [1], cardiac pacemakers [2], prosthetic joints [3], dentures [4] prosthetic heart valves [5], implanted lenses [6], cerebrospinal fluid shunts [7], intrauterine devices [8], breast implants [9], biliary tract stents [10], vascular prostheses [11], penile prosthesis [12], left ventricular assist devices (LVADs) [13], trauma hardware [14] and voice prostheses [15]. Foreign bodies provide a suitable environment for attachment and growth of biofilm that leads to reduced sensitivity to antimicrobial agents as well as the host’s own immune response. As such, approximately 50% of nosocomial infections are associated with indwelling devices [16]. Biofilm infections can also form on tissue surfaces unrelated to foreign bodies, such as in patients with cystic fibrosis and COPD [17,18], native valve endocarditis [19], chronic sinusitis [20], and chronic (diabetic) wound infections [21].

Once formed, treatment strategies vary, but it is generally agreed that in biofilm-related implant infections, definitive treatment will usually involve surgical removal and replacement of the implant. Depending on the type of implant and risk of surgery for the patient, pharmacological treatment alone may be warranted [22]. Yet, the minimum inhibitory concentration for biofilms (MICB) and minimum bactericidal concentration (MBC) of antimicrobial therapy needed to penetrate a biofilm can be difficult to achieve in vivo due to toxicities and side effects incurred at such high drug concentrations [23]. Early biofilms can be treated with an antibiotic regimen alone but can be hard to detect due to the lower sensitivity of routine microbiological examinations for these localized aggregates of bacteria; oftentimes multiple biopsies or proper sonication of the suspected infected area are required to detect the pathogen. These barriers to diagnosis are one reason biofilm is not caught in time for antibiotics alone to be effective [19].

In the United States, revision arthroplasty of an infected joint requires a two-stage revision: removal of the infected apparatus and debridement of infected tissue, and temporary joint fixation for 6–12 weeks while antibiotic treatment is completed. Surgery is then needed to implant a new prosthetic joint. This method, while effective in removing biofilm, has proved to be costly and arduous, oftentimes with a failure rate of up to 22% [24,25]. Most risk factors for failure are those which would not be modifiable for patients, eliciting the need to improve current treatment protocols and options [26]. Multiple surgeries in conjunction with prolonged antibiotic regimens increase the cost of therapy for affected patients [27]. The total projected cost of treating prosthetic joint infections (PJIs) annually in 2030 in the United States will be about $1.85 billion, including $753.4 million for total hip arthroplasty and $1.1 billion for total knee arthroplasty [28].

While the topic of biofilm prevention and removal is one of importance in many areas of clinical medicine, this review will largely cover its place in the treatment of PJI through physical or non-pharmacological methods. Such methods have a long history of use in industrial settings and commonly utilize heat, light, sound, or electromagnetic energy. These methods can potentially be translated to treatment of biofilms on implants within the human body, but so far have remained in developmental stages for treatments of PJI. A recent review summarized novel biological and physiochemical methods of synergistic anti-adhesion composition and antibacterial agents [29]. This review will focus on novel methods of the physical strategies for biofilm disruption and provides a unified summary of the progress that has been made and the challenges that remain.

## 2. Pathogenesis of Biofilms

**Figure 1:**
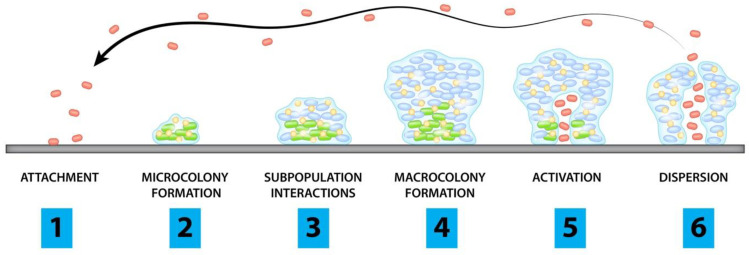
Stages of Biofilm Formation.

In response to a hostile environment, bacteria (and sometimes fungi) form a structure consisting of cells adherent to each other and the foreign surface, surrounded by a self-produced extracellular matrix. This occurs in stages: initially, the planktonic form of the microbial cells will attach to a surface and form microcolonies. They will then form a young biofilm, which differentiates into a structured mature biofilm and will eventually disperse once activated when under stressful conditions (Figure 1) [30].

Stages of biofilm formation:

1. Planktonic cells attach to surface of concern
2. Cells begin to form microcolonies
3. Interactions between subpopulations form microstructures and protective layers
4. Biofilm matures and forms microcolonies
5. Channels form and allow for accumulation of cells
6. Planktonic cells are released from microcolonies

Red dots represent individual planktonic cells while green dots represent biofilm embedded bacteria with blue dots showing protective microstructures

In order to begin adhesion, bacteria secrete extracellular polymeric substances (EPS) consisting of extracellular DNA, proteins, lipids, amyloid fibrils, and polysaccharides such as the polysaccharide intercellular adhesin (PIA) known as poly-*B*(1-6)-*N*-acetylglucosamine (PNAG) in *Staphylococcus* spp. [31]. As bacteria form microcolonies, a hydrogel layer forms that creates a protective barrier between the community and the extracellular environment. The mature biofilm stage is reached as bacteria continue to accumulate into layers of macrocolonies surrounded by channels that help to distribute nutrients and signaling molecules [32] throughout the structure.

Bacteria use quorum sensing (QS) to coordinate gene expression according to their density which functions to regulate the production of virulence factors and create systemic infection. For example, in *S. aureus*, the Spx protein (global regulator of stress response genes) induces expression of the locus (icaR) which negatively regulates the locus for polysaccharide intercellular adhesin (PIA) and icaA (whose product is an N-acetylglucosaminyltransferase that synthesizes PIA oligomers), whereas the Rbf protein (protein regulator of biofilm formation) inhibits icaR, allowing for expression of icaADBC, a locus that regulates PIA expression [33]. In studies of other bacterial species, inhibition of QS receptors such as LasR and Rh1R has provided bacterial hosts with protective effects from biofilm-producing bacteria and reduced QS related virulence during biofilm treatment. Inhibition of the Rh1R target protected human lung epithelial cells from quorum sensing mediated killing by *Pseudomonas aeruginosa* due to treatment with meta-bromo-thiolactone [34]. RNAIII-inhibiting peptide treatment in rats has been found to strongly prevent methicillin resistant *S. aureus* graft infections, indicating the contribution of QS in biofilm formation [35].

Several mechanisms contribute to the antimicrobial resistance of biofilms including low metabolism of antimicrobial agents by cells in the biofilm matrix, presence of persistent dormant cells, and small, highly resistant variant colonies. Stress adaptive responses of the bacterial cells in the biofilm may lead to delayed drug penetration or slow cell growth, to changes in the chemical microenvironment within the biofilm, and to up-regulation of drug resistance genes [36]. Though many strategies related to overcoming biofilm drug resistance mechanisms from a pharmaceutical standpoint have been studied, methods utilizing physical energy have been proposed as alternative solutions. Some have shown promise in either destroying biofilms directly or modifying them to allow for improved effectiveness of pharmaceutical agents.

## 3. Methods of Literature Review

An initial search was run using Ovid MEDLINE using “biofilms” (exp) and “human” (exp) with keyword “removal”. The terms had AND applied and with the search refined to English language results, 590 articles resulted. Additional searches were later conducted on PubMed using search terms “biofilm” AND “removal” AND “joint” AND “human” which produced 82 results. Other PubMed searches of “((biofilm) AND (human) AND (joint) AND (eradication))” returned 67 results. “((biofilm) AND (human) AND (prostheses) AND (treatment) AND (joint))” searched on PubMed yielded 170 results. Relevant resources of selected articles were also utilized as needed.

This search led to articles that either provided a review of novel methods or presented a potential new intervention were included. We focused primarily on non-pharmacological methods of treating PJI biofilms, or on non-pharmacological methods that could be used in a synergistic manner with existing pharmaceuticals. Approximately 125 articles were utilized in conducting this review. Non-pharmacological methods of biofilm treatment fell largely into two groups: Intrinsic methods of preventing the biofilm from forming and extrinsic methods of removing the biofilm.

## 4. Intrinsic Methods

Approaches to intrinsic biofilm prevention can be grouped into a number of strategies that make use of implant modification to prevent or limit biofilm formation. These strategies include co-implantation of an antibiotic reservoir, nanolayer coating of the implant and varying the implant material. These approaches can further be combined such as by varying both material and nanocoating of an implant or by incorporating antibiotics within or in combination with a nanocoating.

Multiple factors, such as surface roughness, may encourage or discourage colonization [24]. For instance, if the roughness profile approaches the size of an individual bacterium (1 µm) it can encourage colonization, while surface pores close to the size of osteoblasts can inhibit colonization. Implant surface porosity can influence fluid flow at the implant surface while high surface hydrophobicity and low surface free energy can also inhibit colonization.

The use of different materials during the revision process presents an opportunity to combat biofilm. Historically, bone cement has typically included antimicrobials as additives to discourage biofilm formation. However, the number of strains of *S. aureus* and *S. epidermidis* resistant to the normally utilized gentamicin and tobramycin is rising. In one study, resistance to gentamicin was 41% and tobramycin 66% [37]. Additionally, implanting a reservoir of antibiotic-loaded bone cement (polymethylmethacrylate, PMMA) is not conclusively proven to ward off infection and can instead create drug-resistant bacteria through prolonged presence of sub-therapeutic concentrations of antibiotic in intra-articular spaces [38]. However, due largely to retrospective case reviews, new material-based strategies are being devised. These include increasing porosity and improving elution profiles of PMMA through modification of its mixing process, using binding agents to increase elution, and using newer antibiotics with longer half-life [24].

Alternative biomaterials to PMMA constitute a possible avenue of exploration but have so far yielded no promising clinical data. Calcium sulfate-loaded, radiopaque beads demonstrated equivalent or better elution characteristics than PMMA, and when impregnated with tobramycin and/or vancomycin were able to reduce *S. aureus* biofilm formation, though they did not reduce biofilms that had already formed [39,40]. Unfortunately, clinical studies using calcium sulfate beads have detailed hypersensitivity reactions in response to the beads, which are not approved for use in the US [24]. Bioactive glass and biocomposites, such as Septacin, a polyanhydride loaded with gentamicin, are considered as biodegradable alternatives [41]. However, these resorbable materials have in general are not validated in clinical studies and cannot keep pace with the growth of surrounding bone, limiting the length of time they provide protection.

A simulation of copper and multi-walled carbon nanotubes by Seo et al. [42] showed some success based on stochastic response that predicted the efficiency of synthesizing nanostructures against *Methylobacterium* spp. The nanostructures increased antimicrobial activity against biofilm in this simulation without increasing toxicity to human cells by damaging the cell wall, causing secondary oxidation of ROS, and releasing copper ions.

Nanoparticles are also a potential source of novel coatings that can be used to prevent biofilm formation. In a study by Gulati et al., titanium nanotube (TNT) arrays loaded with gentamicin were adsorbed to titanium wire by electrochemical anodization. The TNT arrays exhibited a two-phase release of gentamicin, with an initial burst release (37% of weight) followed by a slow release with zero order kinetics over 11 days (Gulati et al. [43]. While the release dynamics of the TNT arrays were observed alone, these nanostructures could potentially be studied within the format of a bone fixative tool or as part of a bone implant assembly. This method shows some promise as an alternative to conventional implants with an added drug eluting component that could prevent biofilm formation and infection.

Silver nanoparticle coatings are one of the most commonly used non-antibiotic antibacterial coatings due to the tendency of silver to leach from the coating and diffuse into bacterial cells resulting in damage to enzymes [44,45,46,47,48,49,50,51]. This nanomaterial has been implemented in a number of ways, including incorporation into hydroxyapatite and chitosan to promote osseointegration, and combined with titanium dioxide to make an anticorrosive material coatings [24]. However, most of these methods are yet to be tested clinically, so potential systemic effects remain unconfirmed [45].

In a study by Zaatreh et al. [52], the viability of a rapidly degrading layer of magnesium on titanium was tested in a co-culture model of *S. epidermidis* and human osteoblasts (hOBs) to verify the antimicrobial and biocompatible properties of the magnesium. When compared to bare titanium Ti6Al4V discs, samples coated with pure magnesium showed an increase in viable hOBs by ~20,000 cells/mL following 7 days of culture. Samples also demonstrated significant bactericidal effect as shown in the reduction of biofilm population by four orders of magnitude after seven days. This study demonstrated the interaction between the implant surface, human tissue, and biofilm and provided an important next step in the development of a potential implant coating that could help prevent and fight biofilm in the short term.

Other coatings have also provided a potential method to prevent or treat biofilm formation, and several approaches including polymers, hydrogels, cyclodextrin, and hydroxyapatite have been tested as drug delivery methods to infection sites [24]. The use of affinity polymers such as cyclodextrin were explored in hernia meshes, vascular grafts, and stents; their utility in orthopedics is better demonstrated when functionalized to hydroxyapatite with extended-release capabilities and osteoblastic cytocompatibility when loaded with tobramycin, rifampicin, gentamicin, ciprofloxacin, vancomycin, or some combination of these antibiotics [53,54,55].

Researchers have also explored polymers that can be refilled with chosen antibiotics rather than preloading a polymer with antibiotics prior to surgical placement [56]. Cyphert et al. designed a polymerized cyclodextrin (pCD) incorporating specific drug affinity that can be placed at the time of surgery and later injected with antibiotics as needed should the patient develop infection. The pCD has hydrophobic inner pockets that take advantage of the affinity interaction with the drug to form a reversible inclusion complex and allow for in situ antibiotic refill and release. In this in vitro experiment, Cyphert et al. utilized rifampicin and minocycline which have relatively high binding energies with β-cyclodextrin and were able to maintain a steady concentration gradient from an agarose tissue phantom into implanted polymer disks. Rifampicin and minocycline were previously shown to be released gradually from a polymerized cyclodextrin that maintains its mechanical integrity under physiological conditions [57]. In the presence of *S. aureus* immature and mature biofilms, the pCD disks were able to be filled with rifampicin and minocycline and showed no statistical difference in affinity and filling when compared to control disks without biofilm formation. Moreover, as rifampicin filling time was increased, after two days the majority of bacteria in a mature biofilm had been eradicated [56]. In the future, this polymer could be formulated as a device coating or formed into nanoparticles to provide a local drug sink that can overcome the delivery issues that reduce the effectiveness of systemic antibiotics in biofilm treatment efforts. Though earlier work has already demonstrated that pre-filled polymers loaded with vancomycin can adequately treat mesh infection in rodents and MRSA hernia defect infections in pigs [58,59], the refillable functions of this polymer have the attraction of individualizing antibiotic treatment to just those who present with infection and can be specific to the bacteria infecting the implant. An area of future study may include the affinity polymer’s ability to be refilled with vancomycin, gentamicin, and other drugs typically utilized to target the tolerant bacteria that so often make up biofilms in PJI. Additionally, though this method is being developed to target hernia mesh infections, its utility for treatment of joint implants in situ will also require additional exploration to determine the ability to reload the polymer once set into a joint environment as well as the polymer’s interaction with the surrounding tissue.

Min et al. [60] proposed a novel method in their development of an implant coated with gentamicin and bone morphogenetic protein 2 (BMP-2) in layers using nanofabrication technology to create conformal nanoscale coatings in a layer-by-layer (LbL) fashion. This LbL assembly facilitated high drug loading while its nanometer to micron scale features allowed for fine tune of multidrug release kinetics at biologically relevant times. This design created a biofilm free environment that encouraged bone growth and repair. The authors demonstrated the capability for antimicrobials to be released over several weeks, while the BMP-2 in underlying layers was sustained longer term, resulting in better bone formation. This specially coated implant was then tested in osteomyelitis-induced mouse models and analyzed by micro-CT. Results revealed that the implants integrated well into bone and showed quantifiable differences in intra-osseous bacterial survival and bone remodeling [60]. Clinical implementation of this technique would allow for a one stage revision rather than the current gold standard of two stage revision, reducing time and costs associated with the lengthy procedure. The success of this implant coating in mouse models may suggest its potential for success in similar layering of artificial heart valves and vascular grafts and could potentially form the basis for a wide variety of coatings in clinical applications.

Another option for coatings is the novel approach taken by Williams et al. [61] in formulating a silicone (polydimethylsiloxane or PDMS) polymer with an active release antimicrobial agent called cationic steroid antimicrobial 13 (CSA-13). CSA-13 is a synthetic analog of naturally occurring antimicrobial peptides. Because it is not a peptide, it is not a target for proteases. The positively charged components of CSA-13 interacted with negatively charged components of bacterial cell membranes, causing membrane disruption and the release of cytoplasmic components [62]. CSA-13 was found to have superior performance over antibiotics and antimicrobial peptides, a longer shelf life and lower cost of production with a broad spectrum of activity and nonspecific method of attack on bacterial cell membranes [61]. Further studies in sheep models with simulated Type IIIB open fractures and mature biofilm inocula demonstrated that CSA-13 reduced the number of bacteria in biofilms to a level that could be addressed by the host immune system. CSA-13 was also found to elute into surrounding tissues and fluids to prevent biofilm-associated osteomyelitis [61,63]. Further sheep modeling in which animals received porous coated titanium implants in the right femoral condyle demonstrated that subjects that received the CSA-13 coated implant prior to a 5 × 10^8^ CFU inoculum of MRSA had no evidence of bacterial infection. In addition, bone growth at the end of 12 weeks was consistent with control subjects that received a non-coated implant and no MRSA inoculum [64]. Williams et al. [65] then utilized their CSA-13 coating against *Pseudomonas aeruginosa* and found an 8 log_10_ reduction in CFU in less than 24 h in a flow cell system. Because these methods focused on treatment of biofilm as opposed to planktonic bacteria, they were not limited by specific MIC guidelines largely developed based on antimicrobial concentrations for planktonic bacteria that would not necessarily translate to biofilm efficacy. In this case, a potentially intrinsic method has shown to be possible not only in preventing biofilm infection, but also in actively treating existing biofilms. Unfortunately, CSA-13 technologies were abandoned with no clinical data, yet N8 Medical licensed other compounds within the same class are being incorporated into Cerashield™ endotracheal tubes and Health Canada granted emergency request use in mechanically ventilated COVID-19 patients.

Williams et al. synthesized a separate class of antibiofilm compounds called CZs (nomenclature based on a company name; Curza) with dual potential to disperse, kill bacteria, and destroy biofilms. Early work shows that CZs can likewise be incorporated into thin film, active release coatings to treat and prevent biofilm implant-related [66,67]. These coating types constitute a significant development in the treatment of biofilm on implants.

A multi-disciplinary biomedical engineering approach was taken by Ehrlich et al. [68] to construct a “smart” novel implant that would prevent biofilm formation. Their approach employed newer technologies that could exploit quorum sensing and had built in antibiotic release. Here, “surveillance” of quorum sensing would in effect “eavesdrop” on the bacteria as they produce intercellular signaling molecules that coordinate metabolic switching and toxin production related to local bacterial burden. In this approach, a microelectromechanical system (MEMS) biosensor sends a signal to a pair of integral gated receptors in response to changes in viscosity resulting from increased concentrations of glucose that occur due to activation of quorum sensing by the bacteria as signified by the production of these intercellular molecules. The receptors then release inhibitory compounds and antibiotics at very high local concentrations. The biosensor and drug reservoir would be connected to a telemetry system accessible to both patient and physician enabling readings of existing conditions on the implant surface. Additionally, the study explored the use of the bioelectric effect in treating PJI with the exposure of biofilm to AC or DC current in the presence of antibiotics. This “smart” implant design shows much potential, yet it is largely untested and would require the support of a manufacturer willing to undergo the prototyping process to develop it for practical use. However, interest in this novel system may be indicative of future design trends that harness the microenvironment inherent to the implant interface itself to prevent the formation of biofilms.

### Conclusions for Intrinsic Methods

A summary of intrinsic methods is provided in Table 1. Questions remain about the implementation of intrinsic methods for biofilm prevention and control. For implants with new coatings, there remains the possibility of damage to neighboring host cells as well as endotoxin release that can follow the death of large amounts of bacteria [62]. Yet, we recognize infection is likewise cytotoxic; a balance must be achieved. We found that in most novel intrinsic biofilm treatments, strategies focused on aspects inherent to successful device implantation–not only must the implant succeed in preventing or treating infection, but it must also allow osseointegration so that it does not detach from the bone over time. In addition, while active coatings are targeted to work on acute infections, passive antimicrobial properties of the implant must last for its lifetime, usually about 20 years for most implants, and so have an increased need for efficacy in treating delayed or chronic infections. There remains a theoretic concern that for any coating that releases an antimicrobial substance that sub-inhibitory concentrations of any given agent could in fact, increase resistance. This poses challenges for developing intrinsic systems that can deliver an antimicrobial compound at adequate concentrations for long periods of time. There may be some role for intrinsic properties of an implant being used to prevent acute infections in the immediate post-surgical period, but this would then necessitate a method that could treat delayed or chronic infection. Novel methods have been proposed that address each of these needs. Yet, while many of them make use of pharmacological and physical methods for both prevention and treatment, there are limited methods available clinically for other uses such as catheters and ET tubes in Canada and Europe; however, no methods have been proven clinically in the US for PJI [69,70]. Thus, we do not yet know the feasibility of implementing these strategies in the clinic, nor the cost of using these new approaches versus cost of current treatment strategies; nor do we know the true longevity of the antimicrobial properties of the implant. However, these emerging technologies may be viable future options to explore and expand on to meet the continuing challenges that PJI biofilms present.

## 5. Extrinsic Methods

**Figure 2:**
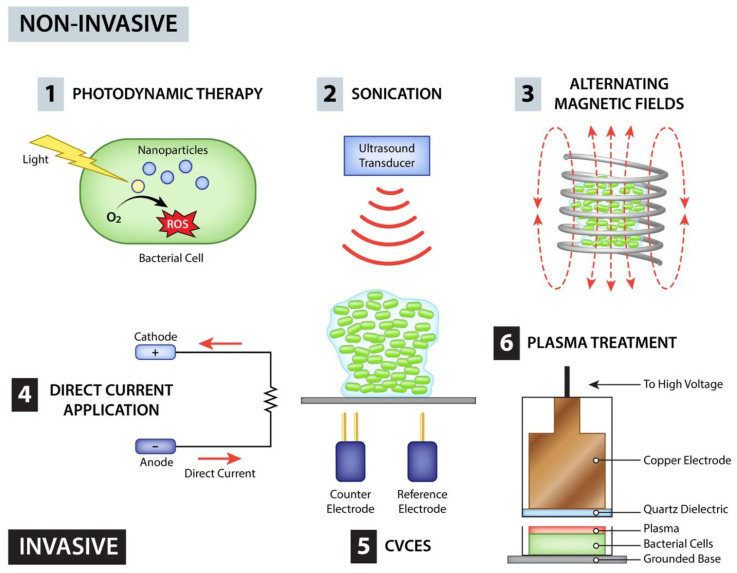
Summary of Extrinsic Methods. Extrinic methods of biofilm destruction can be grouped into two categories, non-invasive and invasive methods. Non-invasive methods include (1) photodynamic therapy in combination with a photosenziting substance which triggers release of ROS, (2) sonication, and (3) alternating magnetic fields causing local heating of implant surface while invasive methods include (4) direct application to the implant causing heating, (5) CVCES which utilizes two implanted electrodes plus the implant itself to create a cathodic voltage causing electrostatic repulsion and creation of ROS, and (6) plasma treatment utilizing floating electrode dielectric barrier discharge.

A second major area of research in biofilm treatment is external methods of treating or removing the biofilm from the implant after the biofilm has already formed. This review largely focuses on technologies that use primarily physical means of removing biofilm and have been tested either alone or in concert with antimicrobials to produce a synergistic effect. Physical means of removing biofilms can be grouped into several subcategories: light-based methods, sound-based methods such as ultrasound or shockwaves, electromagnetic means and plasma or high energy methods of removal as summarized in Figure 2. Based on the mechanism of removal, some physical methods do require accessing the implant while others can penetrate through tissues to reach the implant non-invasively. Because of this, there are multiple factors to consider when evaluating overall effectiveness as well as improvement over current models of treating biofilms.

### 5.1. Photodynamic Therapy

The use of antimicrobial photodynamic therapy (APDT) is studied heavily in the fields of chronic wound care and oral surgery [71,72,73,74,75,76]. The process involves a laser or other visible light emitting source is used in combination with a bactericidal dye or detergent–such as methylene blue, toluidine blue, or sodium hypochlorite–that acts as a photosensitizer (PS). Energy transfer from the photosensitizer to oxygen under light excitation produces ROS that lead to cell membrane lysis and protein inactivation [77]. In these scenarios, APDT was an effective antimicrobial strategy in treating biofilm. The use of photodynamic therapy is studied less directly in PJI, so its benefit to prosthetic joints have to be extrapolated. Vassena et al. [78] showed the ability of APDT with a novel PS, RLP068/Cl, to reduce biofilm on prosthetic material. However, in vivo data with APDT in PJI is scant. An invasive approach would be needed in which the area is surgically opened to apply the photosensitizer to the biofilm to gain access for excitation by the photodynamic light source, and there is the consideration that the photosensitizer of choice must be a specific dye or detergent. However, this technique allows for the use of powerful bactericidal ROS to work against the biofilm. If such a strategy could be developed for use in human patients, it would only require a single stage revision, which would greatly improve on the current two stage revision protocols.

### 5.2. Sonication

Sonication as a treatment modality has progressed mildly in recent years. Sonication has evolved from a treatment method in dentistry and soft tissue infections into an in vitro diagnostic method used in orthopedics to identify PJIs. The utility of sonication stems from its ability to produce cavitation, or the growth, oscillation, and collapse of bubbles in a medium that can produce high energy mechanical effects, and capability to deliver the energy through intact skin from external devices [79]. The collision of a sonic wave with a liquid medium creates regions of alternating compression and expansion-induced cavitation, leading to the formation of gas bubbles. As these bubbles expand and contract, the larger surface area of bubbles in the expansion phase causes gas dissolved in the liquid to diffuse into the bubbles. At a tipping point, the ultrasonic energy is not enough to retain the vapor phase of the bubble and rapid condensation occurs, creating shock waves upon collapse of the gas bubbles [80]. The development of cavitation in a medium is dependent on its dissolved gas, hydrostatic pressure, specific heat of the liquid, the gas in the bubble, the tensile strength of the liquid, and temperature [80]. Antimicrobial mechanisms under these conditions include fatigue of the bacterial cell wall resulting in its damage, intracellular shear forces within bacterial cells induced by microstreaming, and chemical attack from the formation of free radicals during cavitation which weaken the cell wall to the point of disintegration [81]. Low frequency is defined as 20–200 kHz whereas anything greater than 1 MHz is considered high frequency and can either be applied continuously or pulsed [82]. Continuous application of ultrasonic energy can cause the target to absorb the energy and lead to heating, forming the basis of many high intensity ultrasound applications in tissue or bone ablation [83].

Sonication of explanted hardware shows promise in the diagnosis of PJI. Though clinical presentation, joint fluid cell count, imaging studies, histopathology, inflammatory markers, and microbiological assessment are conventionally used to diagnose PJI, sonication results in a higher diagnostic yield. The prosthetic component is typically removed, vortexed, sonicated, and then vortexed again, disrupting the biofilm from the prosthetic surface and producing higher CFU yield in cultures [84]. This strategy shows particular benefit during removal of spacers in second stage revision surgery in detecting subclinical infection [85]. A meta-analysis of 12 clinical trials showed a pooled sensitivity of 0.80 (95% CI 0.74–0.84) and pooled specificity of 0.95 (95% CI 0.90–0.98) in detecting subclinical infection, but limitations included a heterogeneous patient cohort and the use of PJI definitions which deviate from the MSIS consensus criteria [86]. Further, level III diagnostic studies using the MSIS definition of PJI demonstrated improved sensitivity through the use of sonication in diagnosing joint infection [87]. When combined with other molecular diagnostic techniques, sonication shows improved sensitivity compared to conventional tissue culture with sensitivity closer to 60.8% [82,84]. Limitations include determining a normal limit of cell counts that would signify an asymptomatic colonization vs. what level of elevation would constitute a PJI, effect of sonication processing on bacterial metabolic activity and proliferation during bacterial culturing, and difficulties in species identification due to variations in biochemical reactions and phenotype found in sonicated specimens of sessile bacteria [82]. Though the increased ability to more accurately identify an infectious agent may not completely remove the need for surgery, it would help to identify the causative agent of infection earlier and more accurately to better identify subsequent therapeutic approaches. Taking these points into consideration, the use of sonication in diagnosing PJI of prosthetic components that may have suspected or proven infection but have negative conventional aspiration cultures or have undergone preoperative antibiotic administration, shows great promise in improving existing protocols for treatment of PJI.

The action of sonication in treating PJI relies heavily on three separate effects including direct action on bacteria, synergistic effects with antibiotics, and release of antibiotics from bone cement. In vitro data show conflicting results, with some studies noting no antimicrobial effects and others reporting reduced bacterial viability especially towards Gram-negative bacteria; still others have shown biofilm destruction but not eradication or have shown some remaining viability following high intensity ultrasound that destroys the biofilm almost completely [88,89,90,91,92,93,94]. Moreover, the complex interaction of acoustic energy with variations in tissue and implants may be a strong contributor to a lack of consensus results, which is a drawback when comparing multiple studies. In short, work does not yet conclusively show the effectiveness of sonication alone in completely eradicating infection and preventing its recurrence. In addition, damage to nearby cartilage due to sonication was investigated. Reduction in articular cartilage thickness and increased surface roughness of implant components due to sonication, as well as resultant decreased implant longevity were side effects of sonication used for treating PJI [89]. There is also a consideration as to whether sonication of biofilms would cause systemic release of viable bacteria, especially given that results have been mixed as to whether biofilms can be completely eradicated with this method. Further study will need to be conducted to determine if this is potential side effect of biofilm sonication and to ensure the safety of this method.

### 5.3. Plasma Treatment

Another possibility for the treatment and removal of PJI associated biofilm is the use of high energy plasma to destroy the biofilm embedded bacteria; Joshi et al. [95] explored the use of dielectric-barrier discharge plasma. The apparatus was designed to operate in room air and generate microsecond duration high voltage with pulsed cold plasma between a quartz-coated electrode and the sample-carrying surface–in this case the joint implant surface of interest–which also served as a second electrode. The second electrode was not grounded and remained floating (i.e., had a high capacity for charge storage) to ignite discharge plasma when the first electrode approached the surface. Bactericidal effects came from charged and neutral active species generated by the plasma, such as ozone, nitric oxide, superoxide, hydrogen peroxide, singlet oxygen, OH radicals, ultraviolet radiation, electrons, and other charged species [93]. Previously, the floating electrode dielectric barrier discharge (FE-DBD) had been demonstrated for safety on delicate surfaces and animal skin, such as that of mouse and rat due to its micropulse format, which assured that surface temperatures did not rise above 40 °C [96]. Joshi et al. evaluated the effect of their apparatus on both planktonic and biofilm variations of *E. coli*, MSSA, and MRSA via quantification of viable cells using LIVE/DEAD and XTT assays and by colony count assays. Direct plasma was utilized by directly exposing the surface of interest to plasma, which was discharged with an air column of approximately 3 mm between the first electrode and the sample carrying surface, which caused a significant flux of charges to the surface. Indirect plasma was utilized by exposing 100 uL of a fluid of interest to the plasma being discharged, and 80 uL of the fluid being immediately applied to the surface of interest. This method utilizes mostly uncharged atoms and molecules that are generated in plasma and delivers them via gas flow through the plasma region to the surface of interest. The authors found that the degree of surface decontamination was proportional to duration of plasma exposure and that time to plasma mediated sterilization was proportional to bacterial load. XTT assays demonstrated that although resistance to plasma treatment varied by species and strain, all biofilm forms of all pathogens were sterilized in under 120 s, regardless of the use of direct or indirect plasma application, though direct plasma had a more pronounced killing effect. In planktonic form in liquid cultures of 10^6^ to 10^9^ CFU/mL, MRSA strains had more resistance, with sterilization approximately 150 s while other biofilm forms took <120 s. Jet plasma has been utilized in point of care treatment of burn wounds, and the relative safety of dielectric nonthermal plasma and the relative ease with which it can sterilize both dry surfaces and biofilms holds great potential for its clinical use in biofilm removal from implants, thereby reducing painful sequelae and cost from a PJI [97,98,99,100,101]. Interestingly, due to its use of electrode arrays, dielectric plasma presents a better configuration for sterilizing larger surface areas, such as biofilms on implants or other medical devices versus jet plasma, which is a much more focused area of action [96]. More study is required to better quantify the surface area limitations of dielectric plasma technologies with regard to clinical use on implants. If this technology can be implemented to remove biofilm during surgical access to the implant, the need for 2-stage revisions can potentially be reduced. However, invasive intervention to fully access the site would still likely be necessary, and efficacy would need to be proven for surfaces embedded within bone or that are otherwise difficult to access while the implant is in place.

### 5.4. Electric Fields & Currents

Electric fields and currents have been an object of study for their bactericidal effects for some time, as they have a strong influence on the growth or death of both prokaryotic and eukaryotic cells [102]. Biofilm disruption results from a number of factors, including interference of necessary bacterial cellular organization, function, and communication [103,104] as well as bactericidal effects as caused by demonstrated toxicity in salt solutions and synthetic urine [105,106]. These bactericidal effects are hypothesized to be dependent on the formation of toxic substances as a result of electrolysis, oxidation of enzymes and coenzymes, membrane damage, and decreased rate of respiration [107]. In wound care therapy, the field of electroceuticals is explored as a potential avenue for ensuring the healing of wounds and burns. A clinical trial for an FDA approved electric field wound care dressing is underway, but direct electrical stimulation in wound care shows mixed results [104].

A study by Brinkman et al. [108] showed that the electricidal effect of DC was largely based on the generation of ROS as opposed to detachment of the biofilm. When use of the DC current was combined with supplementary antioxidants such as catalase, mannitol, and tempol, the amount of cell death in the biofilm was greatly reduced. These effects, alone or in concert with antibiotics, present an opportunity for the development of new methods with which to treat biofilms. Schmidt-Malan et al. [109] demonstrated that the electricidal effect was effective in either intermittent or continuous form against multiple Gram-positive and Gram-negative species.

Questions arise relating to the use of electric currents to stimulate biofilm detachment, not only around the efficacy of the technique, but also over whether the use of direct current (DC) or alternating current (AC) would be most effective. Previous studies by Costerton et al. and McLeod et al. demonstrated that the use of DC could potentiate tobramycin which resulted in a significant reduction in *Pseudomonas* biofilm [110,111]. The “bioelectric effect” results from the combined use of biocides and antibiotics with a low-intensity DC electric field and takes advantage of the ability of the electric field to reduce the capacity of the biofilm to bind the antibiotic, increase permeability of the membrane, augment transport of antimicrobials, increase bacterial growth and therefore susceptibility to antimicrobials, potentiate oxidants, increase transport through electroosmosis (physical removal of the biofilm through electrolytically produced bubbles) and enhance susceptibility of the biofilm to local temperature increase [102].

del Pozo et al. [112] sought to determine whether the bioelectric effect was generalizable across multiple species of bacteria when combined with most antimicrobials. Pickering et al. [113] previously showed that there was no significant bioelectric effect with vancomycin and *S. epidermidis* while Jass and Lappin-Scott [114] found that the bioelectric effect also did not occur with piperacillin and *P. aeruginosa*. However, del Pozo et al. had found that some combinations demonstrated a significant effect, such as vancomycin with MRSA biofilm and daptomycin and erythromycin when used to treat *S. epidermidis* biofilm, while others did not [112]. These varied results imply that protocols specific to a bacterial species and antimicrobial regimen might have to be developed.

In other studies, Caubet et al. [115] confirmed a bioelectric effect in *Escherichia coli* biofilm when used with gentamicin and oxytetracycline, when using radiofrequency AC (10 MHz). The mechanism behind that bioelectric effect was theorized to differ from that shown with direct current. Radiofrequency electric current does not transport any existing ions as the frequency is such that charged particles vibrate around a mean position and at the intensities used, does not create new ions or any electroporation effects. It also does not produce free oxygen or a major heating effect. Instead, the study theorized that the alternating polarity of the electromagnetic field would vibrate polar molecules and therefore increase fluidity of the charged particles within the EPS. Increased fluidity of the matrix would allow for greater penetration of antibiotics, thereby replicating the bioelectric effect but through a different mechanism.

However, with regard to alternating electrical current alone, Poortinga et al. [116] demonstrated that block current (an alternating current that takes a block waveform as opposed to the more traditionally used sinusoidal waveform) was best for detaching initially adherent bacteria and so surmised that parallel forces were better than perpendicular ones in causing bacterial desorption based on the electroosmotic properties of the fluid flow. Similarly, van der Borden et al. [117] studied the use of both DC and block currents of 60 and 100 uA in detaching *S. epidermidis* from surgical stainless steel and found that DC was more effective in removing biofilm bacteria than block currents. This most likely occurs due to the lack of electroosmotic fluid flow that can occur in the gel-like EPS matrix that prohibits the movement of hydrated ions. Moreover, the higher detachment percentages caused by DC currents over block currents was more likely due to a decrease in the Gibbs interaction energy. Additionally, they found that electrical resistance remained the same throughout the experiment, even after the detachment of the bacteria, and thought that most likely the EPS matrix created most of the electrical resistance.

Ehrensberger et al. [118] described the effects of applying cathodic voltage-controlled electrical stimulation (CVCES) in a three-electrode system to commercially pure titanium substrates (cpTi) with preformed MRSA biofilm on both titanium metal coupons and in in vivo studies wherein cpTi implants were inserted in the humerus of rats. They found that CVCES of −1.8V for 1 h on the in vivo tissue reduced MRSA CFU by 87% in bone and on the cpTi implant by 98%. Based on these results, they reasoned that the CVCES stimulation may have increased interfacial capacitance and decreased polarization resistance of the cpTi, electrical effects that could be associated with the antimicrobial effects of the cpTi. Additionally, passivation of titanium creates a protective oxide layer on the outer surface of the metal that protects the bulk metal while leaving it quite active and responsive to cathodic voltage. This oxide layer can be modelled as a parallel plate capacitor: with applied potential, excess negative charge accumulates at the electrode interface. Because many types of bacteria have a negative surface charge, an electrostatic repulsion force may have stimulated the detachment of bacteria from the metal. Additionally, local reduction reactions at the cathode may have involved local consumption of oxygen, generation of hydrogen and ROS, and increases in pH, all of which could have contributed to the reduction in bacteria. Furthermore, the faradaic-induced change in local pH may have altered the charge distribution and electrostatics within the attached biofilm. Although such local electrical effects may have caused the MRSA reduction, no histological changes were noted on nearby tissue and bone. This study emphasized that due to its three-electrode design, it could maintain constant voltage stimulation with precise control via the minimally invasive placement of an electrical lead to the implant (which would be physically similar to the placement of a surgical drain) with two conductive silicone skin electrodes to complete the configuration.

In further investigations by Nodzo et al. [119], rats that received CVCES in combination with vancomycin were compared to those that received CVCES alone or vancomycin alone. One week after stimulation, animals that received both CVCES and vancomycin had reduced CFU from the implant, in synovial fluid, and bone by 99.8% compared to controls and those that had received CVCES alone. When compared with subjects that received vancomycin alone, the CVCES and vancomycin protocol reduced CFU by 94% on the implant but showed no difference in reducing CFU on the bone and in synovial fluid. No deleterious effects of treatment were noted on histopathological samples of the bone. An additional study [120] then tested prolonged vancomycin given subcutaneously twice daily for 5 weeks and noted that subjects that received both stimulation (either one or two treatments) and vancomycin reduced MRSA bacterial burden from about 10^3^–10^5^ CFU/mL to 0 CFU/mL in comparison to either vancomycin alone or the control treatment. This further supported the results of their earlier study that CVCES combined with vancomycin produced a synergistic, time dependent effect that significantly reduced biofilm burden on implants compared to vancomycin or CVCES alone.

CVCES was also found by Canty et al. [121] to have a preventative effect on both biofilms and planktonic bacteria, first alone at higher voltages (−1.8 V) at longer exposures (8 h), demonstrating the time and voltage magnitude dependent utilization of CVCES. Lower voltage CVCES (−1.0 and −1.5 V) utilized for prolonged exposures (24 h) also showed 3–5 log CFU reductions when used preventatively against MRSA but did not show the same results with *P. aeruginosa*, implying that the effect is more dependent on bacterial cell wall structure than previously thought [122]. Additionally, when combined with vancomycin for MRSA and gentamicin for *P. aeruginosa*, CVCES at −1.5 V for 24 h was able to prevent formation of MRSA and *P. aeruginosa* to below detectable levels, and −1.0 V reduced MRSA to 10^2^ CFU/mL and *P. aeruginosa* to below detectable levels [122].

This highlighted that when combined with antibiotics, CVCES created a synergistic effect to achieve the same or better reductions or prevention of biofilm with lower voltages. Moreover, they were able to utilize the MIC of the antibiotics used, which shows promise over other previously studied methods of electrical stimulation that utilized higher concentrations of antibiotics. Interestingly, Canty et al. also described an alkaline pH shift due to CVCES that achieved a better microbial effect than chemically titrated alkaline pH. They proposed that the electrochemically achieved alkaline pH, which is necessarily combined with other electrochemical processes such as ROS formation, was key to achieving the antimicrobial effect. Researchers thought that the alkaline environment generated by CVCES and its electrochemical processes worked to either increase the effectiveness of the antibiotics or altered the physiology of the biofilm and/or the bacteria itself, allowing for the synergistic effect of CVCES used with antibiotics [122].

Therefore, it is possible to conclude that CVCES could eradicate or prevent biofilms and planktonic bacteria at either high voltage short duration courses, or at smaller magnitude voltage in combination with antibiotics. Implementation of CVCES would involve a minimally invasive procedure to attach a lead to the implant. Without the need for removal and replacement of the implant and even longer-term antibiotics, this option could potentially provide relief to patients who would otherwise need to endure treatment under current guidelines. Preventative applications of CVCES could also prove instrumental in reducing or eliminating the burden caused by biofilms.

In summary, the basis of the success of electrical current in treating biofilms is dependent on the methods with which it is employed. Though DC may have demonstrated a number of potential anti-biofilm mechanisms, studies point to production of ROS as the main factor in the bactericidal effect. Current-induced ROS on their own can cause bacterial cell death but can also be used synergistically with antibiotics to produce the effect. Additionally, AC of various waveforms allow for reversals in polarity altering the fluidity of the EPS matrix and making the matrix easier for antibiotics to penetrate. As antibiotic penetration is the main mechanism, AC are less likely to produce a bactericidal effect on their own and most likely work best in concert with antibiotics. Conversely, sole application of electrical current in PJI could present several challenges–the matrix of the biofilm would largely remain on the implant and potentially provide a nidus for re-infection. Furthermore, it would be difficult to apply the current effectively without invasive surgery to gain access to the implant. However, this technology could enable one-stage revision, and even cleaning of the implant without removal and replacement. This would still greatly reduce the cost and morbidity associated with the current two-stage revision procedure. Additionally, endowing implants with the ability to produce their own electric fields could help enhance the effectiveness of antibiotics and potentially reduce the need for surgical intervention altogether.

### 5.5. Electromagnetic Fields

Another promising evolving technology is the use of electromagnetic fields that take advantage of the conductive properties of the implant material itself. One such area of focus has been the effect of radiofrequency EM fields and their interaction with implants. Passive implants, such as joint prostheses, pose a unique environment for EM field interaction. In most studies, it has been noted that a conductive object, such as a metal joint prosthetic, may cause local enhancement of EM field strength and thus enhance power absorption, although usually not to such a degree as to cause any remarkable excess heating of greater than 1 °C of nearby tissues. The amount by which the field was enhanced was found to be mostly dependent on the orientation, size, shape, and location of the implant [123]. Additionally, *P. aeruginosa* biofilms were found to undergo significant metabolic and biomass reductions when exposed to static one-sided, static switched, oscillating, and combined static and oscillating magnetic fields. When used in combination with magnetic nanoparticles and ciprofloxacin, magnetic fields could be a promising method to removing biofilms from the surface of joint implants [124].

Likewise, other forms of EM energy can heat the surface of an implant and target both the EPS matrix and bacteria residing within the biofilm. To achieve this effect, high frequency (10 kHz and higher) alternating magnetic fields (AMF) can generate heat locally at the implant surface via magnetically induced eddy currents at the metal surface that rapidly heat the implant [125]. This can be implemented in several ways–as in the work of Coffel and Nuxoll [126], in which a magnetic nanoparticle implant coating was used to conduct induced currents. While this method could produce induction heating on any implant regardless of conductive material, it requires the implant to be pre-treated with the tested coating. Alternatively, if the implant itself is made from conductive metal, coating-free induction is possible. Today, implants are mostly made of titanium alloys, stainless steel, special high-strength alloys, alumina, zirconia, and zirconia toughened alumina, all of which have some conductive ability [127]. However, the use of ceramics and plastics in some portions of the implant (usually the acetabulum) would not be conductive and would therefore not produce induction heating. The use of high frequency alternating current can also exploit the skin effect, which restricts current to the outer surface of the conductor. As maximum current density is located at the surface of the metal, it is coincident with the biofilm on implants generating the greatest energy there [125]. A principal advantage of this approach over previously mentioned electrical approaches is that it can be applied to an infected metallic implant non-invasively since magnetic fields can penetrate tissue without attenuation. In fact, in testing titanium alloy (Ti6Al4V) cylinders infected with various pathogens (*S. epidermidis*, *S. aureus*, *P. aeruginosa*, *Bacillus cereus*, and *Candida albicans*), when exposed to pulsed electromagnetic fields causing induction heating, at 60 °C or higher there was a 6-log reduction of CFU or higher for each organism. This demonstrated in vitro that induction heating of a metal implant could noninvasively help to reduce bacterial loads, offering the potential for a new treatment modality for metal-associated biofilm.

Most modeling of induction heating has been based on uniformly shaped metal pieces, but few joint implants have a uniform shape. When used with induction heating, this can lead to nonuniform heating of areas of the implant, and through conduction of that heat, damage to surrounding tissues. Pijls et al. [128] studied the use of segmental heating to selectively heat specific areas of an implant such as in a total hip prosthesis. This method had the advantage of using non-heated metal to act as a heat sink and protect nearby tissues from overheating while facilitating more homogeneous heating of the metal implant. In animal models, this effect can be used to protect surrounding tissue and bone from thermal damage despite rapidly reaching elevated temperatures [129,130].

In addition, Pijls et al. [131] found that induction heating at approximately 60–65 °C or higher for 3.5 min followed by 24 h of vancomycin and rifampicin treatment resulted in total eradication of both young and mature *S. epidermidis* biofilm grown on Ti6Al4V coupons. Total eradication of an *S. aureus* biofilm grown on Ti6Al4V coupons was observed at 80 °C for 3.5 min after 24 h and with the addition of N-acetylcysteine (NAC), total eradication was observed at 60 °C for 3.5 min [132]. With total eradication arises the possibility that patients in need of treatment for PJI could undergo a noninvasive procedure to inductively heat the implant in combination with antibiotics while still safely preserving nearby tissue and forego the need for two-stage revisions altogether.

To more specifically target biofilm associated metal implants, Chopra et al. [133] applied high frequency AMF with a solenoid coil to biofilm-coated washer samples located within the solenoid. This setup induced heating on the washer surface that reached a steady state temperature of 90 °C. For both *P. aeruginosa* and *S. aureus*, a greater than 3 log_10_ reduction in CFU occurred after 5–7 min of AMF. Analysis of the sample washers with scanning electron and confocal fluorescence microscopy revealed that the first minute of AMF seemed to remove the EPS components while 3–5 min of exposure caused a time-dependent reduction in the number of bacteria. This result differed from the results obtained by van der Borden et al. [117] in their use of DC and alternating block currents to detach *S. epidermidis* from surgical stainless steel, in which they found that bacteria detached from the steel prior to the EPS matrix. However, this difference remains consistent with the latter’s hypothesis that the overall negative charge of the bacterial cell wall creates a repulsive electrostatic force detaching bacteria first. In contrast, Chopra et al. take advantage of the local heating effects of alternating magnetic fields, finding that the EPS matrix was affected prior to the bacteria.

Chopra et al. then studied the application of AMF to human-sized implants. To model the safety and efficacy of AMF, 3D CAD images of prosthetic knee joints were analyzed via finite element analysis. One simulated exposure of 600 W AMF resulted in non-uniform heating across the implant and temperatures that exceeded the safety threshold for nearby soft tissue. Next, they tested a protocol in which repeated short duration exposures at higher power (1500 W) were each separated by a delay, referred to as intermittent AMF. This protocol resulted in much more uniform heating while reaching therapeutic temperatures across the model implant in a shorter period. To assure the safety of their apparatus, they also developed a wireless remote acoustic sensing method to detect boiling at the interface between the metal implant and surrounding soft tissue Cheng et al. [134]. Simulations and in vivo studies showed that acoustic emissions were reliably identified at the point of boiling in both tissue mimicking studies and in mouse studies. They suggested that more sophisticated algorithms could be developed that would accurately detect boiling over background noise in a variety of media and would be able to trigger a system to shut off power. Additionally, remote acoustic sensing could enable the use of AMF in implants made from a wide variety of metals, as substantial variability in the resistivity of available materials means heat transfer for different implants may occur at very different rates. By using acoustic sensing to monitor surface temperature for the point of boiling, safety could be assured in unique material implants.

AMF may also have potential application in heat-triggered technologies. Munaweera et al. [135] explored the use of temperature-sensitive liposomes containing ciprofloxacin for temperature-mediated antibiotic release. The liposomal form did not inhibit the release or efficacy of ciprofloxacin as the CFU reduction was similar between low temperature sensitive liposomes (LTSL), high temperature sensitive liposomes (HTSL), and the free form of the antibiotic. Most importantly, however, was the synergistic effect between AMF and the liposomal ciprofloxacin, as the AMF rise in temperature induced the release of ciprofloxacin, resulting in a further 3 log_10_ reduction in CFU of *P. aeruginosa* biofilm compared to AMF alone [135]. This combined overall approach has been shown to be both safe and effective. As one of the few non-invasive methods described, it could potentially lead to the avoidance of surgical revision, resulting in a reduction in the morbidity and costs currently associated with treating PJI.

### 5.6. Summary of Extrinsic Methods

Once a biofilm has formed on an implant, drug tolerance increases and makes it difficult for most conventional pharmacological treatments to penetrate and effectively treat the infection. Non-pharmacological methods usually utilize some form of physical or chemical agitation of the biofilm to either directly cause bacterial death or to increase antibiotic access to the infected area. These methods also have the advantage of not being reliant on bacterial metabolism and to some extent, drug delivery to the infected area. Because these devices make use of physical energy to disrupt the biofilm, they are not limited by some of the toxicity liabilities that various pharmaceuticals might have. They are also not limited by the lifetime of the implant itself and so can be utilized regardless of the chronicity of the infection. A summary of extrinsic methods is available in Table 2.

However, the lack of established safety standards does highlight one of the drawbacks of non-pharmacological methods of biofilm treatment–therefore, testing to ensure the safety and integrity of nearby tissue and bone is paramount to ensuring the proper utilization of these various approaches. While a certain threshold of energy must be reached to cause a significant antimicrobial effect, dependent on the mechanism of action of the method, this must be balanced against preventing damage to local tissue and may require some element of safety monitoring to ensure the proper operation of the device or method. Most studies have only observed these effects in vitro, and while some have conducted in vivo observation of local toxicities, making the jump to clinical application will require further investigation to assess both short and long term safety effects.

One method to limit potential toxicity of both physical and pharmacologic methods for treating biofilms is to use both in concert to maximize the antimicrobial effects while minimizing dosages of each treatment modality. Such synergy is proposed to be achieved when one eradication method acts on the implant surface while another acts on the outer border of the biofilm and progresses to the biofilm-implant interface, allowing for a bidirectional approach [132]. Another proposed mechanism is that the physical means of removal acts to alter the physiology of the biofilm or even the bacteria itself to allow for better penetration and activity of the antibiotic [81,110,111,112,115,122]. However, the mechanisms of synergy that utilizes physical means of biofilm removal have not been fully studied. Regardless, this phenomenon has been utilized to great effect by several novel methods for reducing biofilm load or obtaining full biofilm eradication while limiting side effects.

## 6. Conclusions

The clinical challenges presented as a result of biofilm formation on prosthetic joints have underscored the need for innovative solutions in this area. Given that current therapies can often lead to recurrent surgeries and increasing morbidity [26], alternative methods to remove biofilm or prevent their formation altogether are desperately needed. Prevention of biofilm formation depends on numerous factors, and many intrinsic methods have been tested that approach the problem in a variety of ways. Future testing in in vivo models and in human trials may reveal which of these methods provides the best results, and could potentially eliminate the need to consider biofilm treatment altogether. However, a number of challenges remain with intrinsic methods, particularly the ability to maintain this anti-biofilm effect for long periods of time. In contrast, challenges of the extrinsic methods include a number of techniques that will require direct contact with the implant. However, if the biofilm can be removed in one surgery, especially in a minimally invasive procedure, this type of approach would still be a great improvement over existing protocols. Those methods that allow for a non-invasive approach in treating the biofilm have the greatest potential for change to present day treatment guidelines, and therefore hold much promise. There may even be a future for preventative intrinsic methods and extrinsic treatments to work in concert. The hope is that one or more of these new technologies will provide numerous benefits to future patients in prolonging the useful life of their implant.

## Figures and Tables

**Table 1:** Intrinsic methods. Methods that make use of physical, chemical or biological aspects of the implant itself to prevent or reduce biofilm formation and growth.

Method	Site of Implant Modification	Strategy/Approach (Biological, Chemical, Physical)	Advantages	Progress towards Clinical Use/Disadvantages	Refs.
Bioactive glass and biocomposites	Material	Drug loaded, biodegradable		Evidence of clinical efficacy lacking	[24]
Mixing process modified bone cement (PMMA)	Material	Drug loaded reservoir	Improved elution profiles for better delivery of reservoir of antibiotic	Can create drug resistant bacteria; randomized control trials ongoing	[24]
Calcium sulfate loaded radiopaque beads	Surface	Drug loaded reservoir	Improved elution profiles to PMMA	Cannot reduce already formed biofilms; can induce hypersensitivity reaction	[40]
TNTs with 2 phase release	Surface modification/nanoparticle coating	Drug carrier for local delivery	Release of ROS increased antimicrobial activity	In vitro study	[42,43]
Mg on Ti	Surface coating	Release of Mg ions created bactericidal alkaline environment	Use of ROS without harm to nearby osteoblasts	In vitro study, limited to 7 days of culture	[52]
Ag nanoparticle coating	Coating	Bactericidal through release of biologically active ions, creation of ROS, interaction with sulfhydryl groups	Can be incorporated into a number of materials	Some in vivo work completed	[44,45,46,47,48,49,50,51]
Poly-cyclodextrin in situ antibiotic treatment	Implant coating or drug delivery device	Polymer with drug affinity for loading and release	Refillable; can be both preventative and therapeutic	In vitro study in hernia mesh; not yet explored specifically for PJI	[55,56,57,58,59]
LbL drug loading	Coating	High drug loading, encourage bone growth, repair. Timed multidrug release.	Encourages bone growth and repair	Studies in rats; would require one stage revision	[60]
PDMS with CSA-13	Synthetic analog peptide coating	Drug loaded, cationic interaction with neg charged bacteria.	Avoids protease degradation. Both preventative and therapeutic	Studies in sheep with recent emergency use in ET tubes	[61,62,63,64,65,66,67]
Polymers, hydrogels, cyclodextrin, and hydroxyapatite	Material/coating	Drug delivery	Some materials have extended release properites	Cyclodextrin coated meshes have progressed to in vivo animal studies	[24]
“Smart” implant through monitoring of quorum sensing activity	Built in MEMS biosensor	Exploits quorum sensing, antibiotic release, telemetric control	Antimicrobial properties built into implant, would not require additional revisions	Needs substantial support from manufacturer to prototype for practical use	[65]

**Table 2:** Extrinsic methods. Methods that make use of external energy sources to treat biofilm.

Method/Strategy	Physical Effect	Bactericidal Effect	Advantages	Progress toward Clinical Use/Disadvantages	Refs.
**Photodynamic Therapy**					
Laser excitation of PS	Energy trans photosensitizer → O_2_	ROS generation	Acts directly on bacterial biofilms	Not yet advanced to in vivo trials; invasive procedure needed to access implant	[71,72,73,74,75,76,77,78]
**Sonication**					
Cavitation oscillation-driven rectified gas diffusion, micro-streaming, bubble collapse, ROS formation		bacterial cell wall fatigue, micro-streaming induced intra-cellular shear forces, ROS attack	Acts directly on bacteria and in synergy with antibiotics	In vitro studies; some evidence of cartilage damage from sonication, lack of consensus results	[88,89,90,91,92,93,94]
**Plasma Treatment**					
Dielectric barrier discharge plasma: ms-high V pulsed cold plasma bt quartz and sample		Generation of bactericidal species: ozone, nitric oxide, superoxide, hydrogen peroxide, singlet oxygen, OH radicals, ultraviolet radiation, electrons	Rapid sterilization, but varies by strain; can sterilize large surface areas	Not advanced to clinical stage; invasive procedure needed to access implant with embedded surfaces potentially inaccessible	[95,96,97,98,99,100,101]
**Electric Fields and Currents**					
Electroceuticals	Formation of toxic substances due to electrolysis	Disruption of internal bioelectric milieu	Can help activate host immune system	FDA approved, clinical trials underway for wound care w mixed results	[102,103,104,105,106,107]
DC current	Bioelectric effect: Reduces biofilm resistance to antibiotics	Electricidal effect of ROS rather than detachment	Bactericidal on its own	In vitro studies with varied results; requires invasive one-stage revision, matrix remains on implant	[108,109,110,111,112,113,114]
AC current	Alternating polarity may increase fluidity of antibiotics	Utilizes electroosmotic properties of matrix to detach biofilm	Easier penetration of antibiotics into biofilm	In vitro studies; requires invasive one-stage revision, only works in concert with antibiotics	[115,116,117]
CVCES	Modeled as capacitor, excess neg charge at interface	Repulsion; creation of alkaline environment	Combined with antibiotics can effectively treat and prevent biofilms and planktonic bacteria	In vivo rodent models; does require minimally invasive procedure	[118,119,120,121,122]
**Electromagnetic Fields**					
Conductive object in magnetic field	Dependent on orientation, size, shape, location	Metabolic, biomass reduction on exposure to static 1-sided, static switched, oscillating, & combined MFs	Non-invasive, works synergistically with antibiotics and NAC	In vivo studies; Non-uniform objects leads to non-uniform heating, requiring heat sinks or segmental heating	[123,124,128,129,130,131,132]
Conductive object in AMF	Eddy current generated induction heating	Heat source in direct contact with biofilm	Non-invasive; AMF uses skin effect and restricts heating to surface	In vitro studies; non-conductive surfaces (plastic, ceramic) untreated	[125,127]
Conductive coating in AMF	Heat generated by magnetic nanoparticles in AMF	Heat source in direct contact with biofilm	Non-invasive; AMF uses skin effect and restricts heating to surface	In vitro study; implant must be pre-treated with coating	[126]
High frequency (continuous or pulsed/intermitent)	Skin effect	Heat source in direct contact with biofilm	Non-invasive, save, effective, synergy with liposomal antibiotics	In vivo animal studies	[133,134,135]

## Data Availability

No new data were created or analyzed in this study. Data sharing is not applicable to this article.
